# Short- and Long-Term Outcomes in Horses Following Laparoscopic Nephrosplenic Space Ablation

**DOI:** 10.3390/vetsci13020196

**Published:** 2026-02-17

**Authors:** Anna Cerullo, Nicola Scilimati, Matteo Riccardo Di Nicola, Luca Colla, Susanna Mazza, Alice Bertoletti, Sara Nannarone, Rodolfo Gialletti

**Affiliations:** 1 Istituto Zooprofilattico Sperimentale del Piemonte, Liguria e Valle d’Aosta, Via Bologna 148, 10154 Turin, Italy; anna.cerullo@izsplv.it; 2 Department of Veterinary Science, University of Parma, Strada del Taglio 10, 43126 Parma, Italy; nicola.scilimati@unipr.it (N.S.); mazza-susanna@outlook.it (S.M.); rodolfo.gialletti@unipr.it (R.G.); 3 Department of Biology, University of Pisa, Via Luca Ghini 13, 56126 Pisa, Italy; luca.colla@phd.unipi.it; 4 Department of Veterinary Medicine, Sport Horse Research Centre, University of Perugia, Via San Costanzo 4, 06126 Perugia, Italy; alicebertoletti1@gmail.com (A.B.); sara.nannarone@unipg.it (S.N.)

**Keywords:** equine, colic syndrome, nephrosplenic space ablation, left dorsal displacement, long-term follow-up

## Abstract

Left dorsal displacement of the large colon (LDDLC) is a common cause of colic in horses and it is frequently associated with recurrent episodes, with significant implications for horse management, athletic performance, and owner satisfaction. Laparoscopic nephrosplenic space (NSS) ablation is a preventive surgical technique aimed at reducing recurrence in horses with a history of medically or surgically treated LDDLC. This retrospective study evaluated 48 horses that underwent laparoscopic NSS ablation in two Italian Veterinary Teaching Hospitals between 2016 and 2024. Short-term outcomes, including immediate postoperative complications and discharge status, were assessed. Long-term follow-up data were collected via owner questionnaires, focusing on survival, recurrence of colic, post-surgical complications, return to athletic or breeding activity, and owner satisfaction. Most horses were discharged without complications (91.7%), while postoperative colic occurred in 8.3%. Kaplan–Meier estimated one-year post-discharge survival was 83.3%, and 81.3% of horses were alive at last follow-up (range 0.7 months to 8.8 years). Post-discharge complications were reported in 31.2% of horses, most commonly recurrent LDDLC. Return to preoperative athletic or breeding activity was reported in 70.8% of cases, and owner satisfaction was high (83.3%). Time-to-event analysis indicated markedly reduced survival in horses requiring laparotomy during follow-up. Bayesian logistic models suggested lower mortality and fewer post-discharge complications in horses with prior colic surgery, whereas a history of recurrent colic and laparotomy during follow-up was associated with higher mortality and complications. These results indicate that laparoscopic NSS ablation is an effective and safe procedure for long-term management of horses at risk of LDDLC, allowing a high rate of survival, functional recovery, and owner satisfaction, despite the persistence of occasional colic episodes.

## Simple Summary

Left dorsal displacement of the large colon is a frequent cause of colic in horses. It can recur, limiting performance and increasing costs and management demands for owners. We evaluated a minimally invasive surgical procedure that closes the space between the spleen and the left kidney to reduce the risk of the colon becoming trapped. We reviewed 48 horses treated in two Italian Veterinary Teaching Hospitals and followed them for up to nine years. Most horses were discharged without early complications, and most were still alive at the last follow-up. About one in three horses had colic episodes after discharge; most often this was suspected to be a recurrence of left dorsal displacement, and only a small proportion required abdominal surgery. Most horses returned to athletic or breeding activity, and most owners reported a positive outcome. Our results support this laparoscopic preventive procedure as a safe option that can help maintain long-term function and welfare. It can also provide reassurance to owners, although some colic episodes may still occur and require monitoring.

## 1. Introduction

Left dorsal displacement of the large colon (LDDLC) is a common cause of colic in horses, with an incidence ranging from 3% to 9% among horses referred for colic [1,2,3,4,5,6]. LDDLC is a non-strangulating displacement of the large colon that occurs when the left dorsal and ventral colons move dorsally and medially, rotating approximately 180° and becoming positioned between the left kidney and the spleen. This condition is associated with intestinal motility dysfunction and tympany [7].

The anatomical configuration of the nephrosplenic space plays a key role in the predisposition to LDDLC. Previous studies have shown that horses with a deep, “V-shaped” nephrosplenic space are more susceptible to displacement than those with a shallow, “L-shaped” space, where the colon can more easily return to its normal position [2,8]. Furthermore, according to Gandini [9], shallow nephrosplenic spaces tend to better respond to medical treatment, while deeper spaces have a higher risk of entrapment that requires surgical intervention.

Although LDDLC is a non-strangulating entrapment, episodes lasting longer than 24 h may lead to colonic congestion, oedema, and damage to the intestinal wall, increasing the risk of visceral rupture or the need for intestinal resection [10]. Additionally, recurrent colic episodes pose a significant concern for horses’ quality of life and management by the owners, with relevant economic and logistical implications.

Clinical examination, rectal palpation, and transabdominal ultrasonography could be indicative of LDDLC, although definitive diagnosis can only be reached through exploratory laparotomy [11,12,13]. In case of colic syndrome related to LDDLC, treatment options include intravenous fluid therapy and controlled exercise, as well as the administration of phenylephrine or the rolling technique under general anaesthesia [14]. Although historically employed, these techniques carry risks and do not always guarantee a successful resolution [15,16]. Controlled exercise is most effective if it is started early, whereas excessive activity in the presence of tympany or impaction can increase the risk of complications, including large colon rupture [17,18,19,20]. Phenylephrine, while useful in reducing splenic volume and facilitating the resolution of displacement, can cause adverse effects such as hypertension, second-degree atrioventricular block, profuse sweating, and tachycardia, in addition to a rare risk of haemorrhage [14]. For these reasons, some clinicians prefer to proceed directly to surgical correction through exploratory laparotomy [3,12,18,21].

Due to the high risk of recurrence, preventive strategies have been proposed, including closure of the nephrosplenic space. This procedure is recommended either in cases of recurrent colic episodes with suspected nephrosplenic entrapment (NSE) or following a diagnosis of NSE confirmed by laparotomy. Several surgical techniques for nephrosplenic space (NSS) ablation have been described [5]. The laparoscopic approach is currently the most widely used technique, with various methods described to achieve NSS ablation [8,22,23,24,25]. Traditionally, these procedures required complex handling and intracorporeal knot tying, with a risk of suture failure even in experienced hands [26].

Historically, NSS ablation was performed via an open left flank laparotomy under general anaesthesia; however, standing laparoscopy is now widely adopted as a minimally invasive option that reduces surgical impact, shortens recovery time, and decreases the incidence of complications.

Even within laparoscopic approaches, several methods have been explored, including internal or external knots, barbed sutures, and prosthetic meshes [27,28]. Traditional sutures may increase technical demands because they require maintaining tension and intracorporeal knot tying, factors that can increase the risk of failure even in experienced hands [23,26]. For this reason, self-retaining barbed sutures are now widely used for standing laparoscopic NSS ablation, as they allow continuous closure without intracorporeal knot tying and may reduce operative time while maintaining satisfactory clinical outcomes [23,29]. Comparative studies have reported shorter surgical times with barbed sutures compared with alternative techniques, with comparable clinical results [29].

Several studies have confirmed the effectiveness of laparoscopic NSS ablation in reducing the recurrence of colic associated with LDDLC [9,30,31], although few have compared results with a population of horses with LDDLC that did not undergo NSS ablation or reported data concerning the long-term follow-up and long-term complications [5,30].

The aim of this study was to analyse short- and long-term survival data of horses undergoing laparoscopic NSS ablation in two Italian Veterinary Teaching Hospitals (VTHs). Additionally, return to sporting activity, general health status, and owner satisfaction following the intervention were assessed.

## 2. Materials and Methods

### 2.1. Study Population

The retrospective study included horses that underwent laparoscopic NSS ablation between 2016 and 2024 in two Italian VTHs. Horses without a complete long-term follow-up report were excluded from the study. Inclusion criteria comprised horses older than one year that underwent NSS ablation following surgical LDDLC or recurrent LDDLC medically treated. For each horse, demographic data were collected, including breed, age, body weight, gender, history of recurrent colic, number of previous hospitalisations for colic, and previous surgical intervention. Details of the surgical procedure, type of suture used, in-hospital complications, discharge outcome, postoperative follow-up, and return to athletic or reproductive activity were also recorded.

### 2.2. Surgical Techniques and Short-Term Follow-Up

All horses underwent the procedure after written informed consent was obtained from the owners. A standing left flank laparoscopy for NSS ablation under sedation via continuous rate infusion of detomidine (0.1–0.5 µg/kg/min IV) with a syringe pump was performed. The left paralumbar fossa was clipped, aseptically prepared, and draped. Local anaesthesia (15–20 mL of 2% lidocaine) was infiltrated into the skin, fascia, and muscle at the three planned portal sites. The first port was placed caudal to the 18th rib using a 10 mm blunt trocar, and a 0°, 33 cm laparoscope was introduced to explore the abdomen. Under direct visualisation, two additional ports were created: one in the 17th intercostal space with a 12 mm trocar and another 5 cm ventral to the first using a 5 mm trocar. The laparoscope was then moved to the second port for the procedure.

The NSS was closed using a continuous absorbable polydioxanone self-retaining barbed suture [Filbloc^®^ (Filbloc^®^ Assut Europe S.p.a., Magliano dei Marsi, L’Aquila, Italy) or Stratafix™ (Ethicon, Inc., a Johnson & Johnson MedTech Company, Somerville, NJ, USA)] or a composite mesh consisting of knitted polypropylene and expanded polytetrafluoroethylene (ePTFE) [(Bard^®^ Composix Mesh, Becton Dickinson Italia S.p.A, Milano, Italy)] placed between the perirenal fascia and the splenic capsule. The suture was initiated at the cranial aspect of the ligament and advanced at regular intervals until complete obliteration of the space, with final reinforcing passes to secure the closure.

As regards the mesh technique, after measuring the distance between the spleen and the perirenal fascia, a composite mesh slightly wider than the gap was selected to minimize tension. The mesh was rolled for laparoscopic insertion and positioned so that the polypropylene surface faced the spleen and perirenal tissues, while the ePTFE side was oriented toward the abdominal viscera. Fixation was achieved using titanium helical fasteners applied at regular spacing [29].

At the end of surgery, the trocars were removed and the muscle fascia was closed with a simple continuous suture of absorbable USP 1 while the skin was closed with a simple interrupted suture of non-absorbable USP 0 nylon thread.

All horses received a single postoperative dose of antibiotics (streptomycin 10 mg/kg body weight, intramuscularly) administered 12 h after the preoperative administration, and anti-inflammatory therapy (flunixin meglumine, 1.1 mg/kg, SID, intravenously) for five days. Clinical status upon recovery from the procedure, postoperative complications (postoperative colic, POC), any relaparotomies performed during hospitalisation, surgical site infection (SSI), and outcome at discharge (discharged alive, in-hospital euthanasia, in-hospital death) were recorded.

The short-term follow-up was defined as the clinical status recorded at the time of discharge.

### 2.3. Long-Term Follow-Up

Long-term follow-up was obtained through telephone interviews with the owners. Data were collected on survival, colic recurrence, any additional surgical interventions, postoperative complications, return to athletic or reproductive activity, and owner satisfaction with the procedure. Owner satisfaction was categorised according to the reported level of satisfaction: responses indicating “satisfied” or “very satisfied” were classified as positive, whereas responses indicating dissatisfaction were grouped as non-positive. Overall satisfaction was assessed considering the horse’s health status, postoperative management, costs, and athletic performance.

Long-term follow-up was defined as the period between discharge and either the date of death or the date of data collection via telephone interview. The questionnaire was initiated in January 2025, and ended in February 2025. For horses that were no longer alive or had been sold, information regarding the date and, when applicable, the cause of death was recorded.

### 2.4. Data Analysis

Signalment data, medical history (including previous colic surgery, history of recurrent colic, and whether the LDDLC was treated medically or surgically), surgical data (date of surgery, duration of hospitalisation, type of suture material used), short-term hospitalisation data, and outcome were recorded. Age and weight data were analysed using the Shapiro–Wilk normality test, and results were reported as mean ± standard deviation (SD) or median (range), as appropriate.

The effects of previous colic surgery and laparotomy after NSS ablation during follow-up on post-discharge survival were assessed separately using Kaplan–Meier estimates and log-rank tests. In addition, Cox proportional hazards regressions were fitted to evaluate the effect of these factors on survival. Deaths were treated as events, whereas horses alive at last follow-up were right-censored; survival time was defined as the number of days from hospital discharge to the last follow-up (15 February 2025 for all live horses). Survival estimation and Kaplan–Meier curve generation were performed with the *survival* [32] and *survminer* [33] packages.

To further investigate factors influencing survival (1 = death, 0 = survival) and potential postoperative complications (1 = yes, 0 = no), two independent Bayesian models were fitted. Bayesian inference was used to evaluate uncertainty in model parameters and predictions by estimating the full posterior distributions and reporting 95% credible intervals and posterior probabilities for clinically relevant effect directions. Outcome probabilities were modelled with generalised linear models (GLMs) assuming a Bernoulli response.

For the survival model, explanatory variables included: (i) previous colic surgery; (ii) a history of recurrent colic; and (iii) whether the horse underwent laparotomy after NSS ablation during follow-up. For the postoperative complications model, laparotomy after discharge was not included as an explanatory variable because it represents a post-discharge complication and would conceptually overlap with the outcome. Weakly informative normal priors were used (μ = 0, σ = 5 for the intercept; μ = 0, σ = 2 for coefficients). Sampling used four chains with 8000 iterations per chain and 3000 warm-ups. Convergence was checked for both models (R-hat = 1 for all predictors). To improve sampling, the maximum tree depth and target sampling probability were set to 15 and 0.95, respectively. Modelling and hypothesis tests on coefficients were performed with *brms* [34]; the half-sample mode (HSM, a measure of central tendency) was estimated with *modeest* [35]; 95% credible intervals were computed with *HDInterval* [36]; and posterior distributions were visualised with *bayesplot* [37]. All analyses were carried out in R 4.4.1 [38].

## 3. Results

### 3.1. Study Population

A total of 48 horses were included in the study between 2016 and 2024. The final dataset included horses with a median age of 11 years (range 1.5–20 years). The most represented breed was the Italian Saddlebred (n = 15; 31.2%), followed by Belgian Warmblood (BWP) and Arabian (n = 6 each; 12.5%), Hanoverian, Holsteiner, and Anglo-Arab (n = 3 each; 6.3%), Zangersheide and KWPN (n = 2 each; 4.1%), and Quarter Horse, Paint Horse, Friesian, Czech Warmblood (CZWEB), Lusitano, Murgese, Oldenburg, and Standardbred Trotter (n = 1 each; 2.1%). A total of 25 geldings (52.1%), 13 mares (27.1%), and 10 stallions (20.8%) were included.

The median number of hospitalisations for colic prior to laparoscopy was 1 (range 1–4). Most horses (n = 28; 58.3%) had a history of recurrent colic, while the remaining 20 horses (41.7%) had no previous episodes of colic. Most horses (n = 30; 62.5%) did not undergo colic surgery before NSS ablation, while the remaining 18 horses (37.5%) had a history of colic surgery.

### 3.2. Surgical Techniques and Short-Term Follow-Up

All horses included in the study underwent laparoscopic NSS ablation. Absorbable polydioxanone self-retaining barbed suture was the suture material used in most cases (n = 47; 97.9%), USP 1 in 14 cases (29.8%), Filbloc^®^ USP 2 in 32 cases (68.1%), and Stratafix™ in 1 case (2.1%). The polypropylene and expanded polytetrafluoroethylene (ePTFE) mesh was used in only one horse (2.1%).

Immediate procedural success (defined as discharge without complications) was achieved in 44 horses (91.7%), whereas 4 horses (8.3%) experienced POC requiring specific management. All horses were discharged from the hospital after a median hospitalisation time of 11 days (range: 3–43).

### 3.3. Long-Term Follow-Up

Most horses (n = 39; 81.3%) were alive at the time of the last follow-up, while nine horses (18.7%) had died by the time of the long-term follow-up. Of these, three were euthanised during laparotomy for colic (one for NSE and two for other types of colic), one died due to haemorrhagic colitis, and the remaining five horses died spontaneously due to colic syndrome.

**Figure 1:**
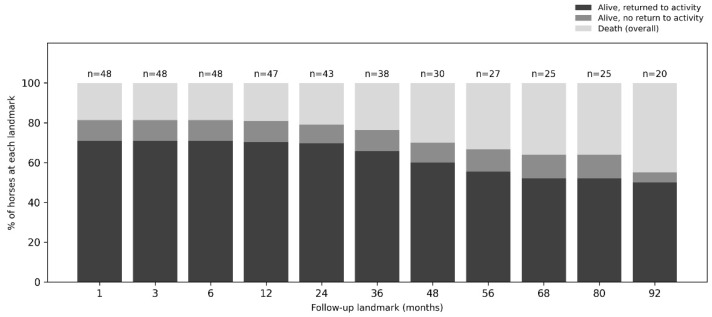
Landmark analysis of long-term outcomes at last owner contact. For each follow-up landmark, percentages are calculated using only horses evaluable at that time point based on the date of last contact; horses that died before the landmark are retained in the denominator.

Long-term follow-up ranged from 20 days to 3197 days (approximately 0.7 months to 8.8 years; median = 1386, IQR = 2026). Because owner interviews were conducted within a defined study window, outcomes were additionally summarised at prespecified follow-up landmarks based on the date of last contact, to minimise bias related to horses that had not yet reached longer follow-up. Overall, 34 horses returned to athletic or breeding activity, five were alive without return to activity, and nine died. At 12 months (n = 47 evaluable), 33 out of 47 (70.2%) horses had returned to activity, five out of 47 (10.6%) were alive without return, and nine out of 47 (19.1%) had died. At 48 months (n = 30 evaluable), 18 out of 30 (60.0%) had returned to activity, three out of 30 (10.0%) were alive without return, and nine out of 30 (30.0%) had died. Landmark-specific denominators and outcome distributions across all time points are shown in Figure 1.

Postoperative complications after discharge were reported in 15 out of 48 horses (31.2%), due to postoperative colic. Recurrence of LDDLC after NSS ablation was reported in nine out of 15 horses with postoperative colic (60%). Among these, five horses out of nine were managed medically (55.6%), while four underwent colic surgery (44.4%). In one out of the 15 horses, gastric ulcers and fever were also reported. Among the patients that underwent surgery after NSS ablation, three procedures were relaparotomies.

**Figure 2:**
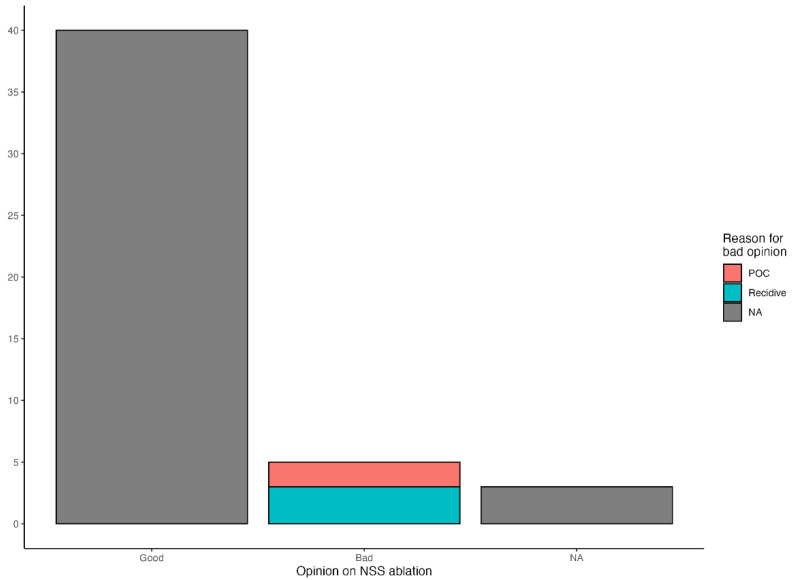
Owner-reported opinion on NSS ablation at long-term follow-up (n = 48). The *x-axis* indicates the owner-reported opinion categories; the *y-axis* indicates the number of owner opinions.

Return to athletic or reproductive activity was reported in 34 out of 48 horses (70.8%). Owners reported a positive opinion of the procedure in 40 cases (83.3%), mainly citing clinical improvement and absence of colic episodes as the primary reasons, while a negative opinion was reported in five cases (10.4%), mainly attributed to recurrence of the condition or the onset of further colic episodes; owner feedback was unavailable in the remaining three cases (6.3%) (Figure 2).

### 3.4. Statistical Analysis

**Figure 3:**
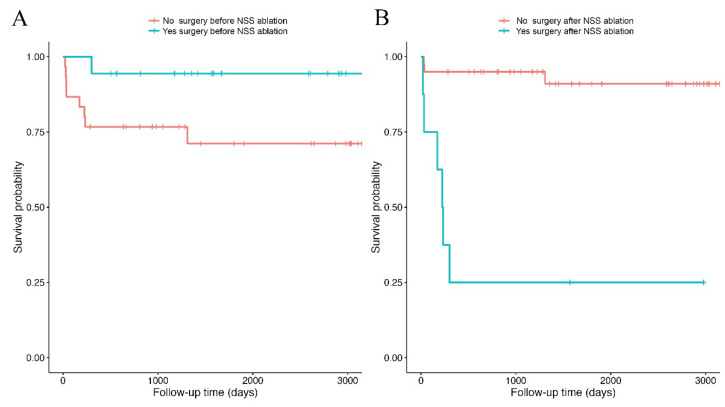
Survival curves computed using the Kaplan–Meier estimator. The curves represent the survival probabilities of discharged horses that did or did not undergo colic surgery prior to the index NSS ablation. (**A**), and horses that did or did not require laparotomy after initial discharge (**B**). Vertical bars on the curves indicate censored observations, and downward steps indicate death events.

The Kaplan–Meier estimated one-year post-discharge survival probability was 83.3%. The log-rank test showed no significant difference in post-discharge survival between horses with prior colic surgery and those without (χ^2^ = 3.4, df = 1, *p* = 0.07; Figure 3A). The Cox model yielded a hazard ratio of 0.18 for previously operated horses, indicating an approximately 82% lower risk of death compared with horses without prior colic surgery (*p* = 0.10). In contrast, survival differed significantly between horses that required colic surgery during follow-up and those that did not (χ^2^ = 25.1, df = 1, *p* < 0.001; Figure 3B). Laparotomy after NSS ablation was associated with an increased risk of death in the Cox model (Hazard Ratio = 15.12, *p* < 0.001).

**Figure 4:**
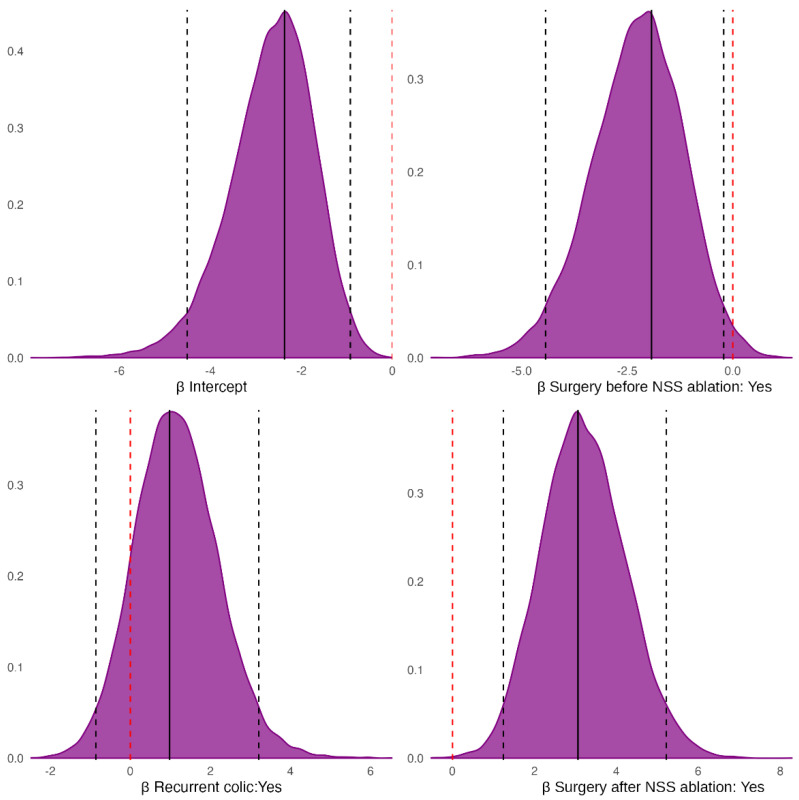
Posterior probability distribution of the fixed-effect coefficients from the Bayesian model for the outcome. Black solid vertical lines mark the half-sample mode (HSM); black dashed vertical lines mark the 95% highest-density interval (HDI_95_); the red dashed vertical line marks zero. Note: the HSM is a robust estimate of the posterior mode [39], whereas the HDI_95_ is the narrowest interval containing 95% of the posterior probability mass [40,41].

The Bayesian model estimated a lower death probability for horses with prior colic surgeries (P_Prior colic surgery < No colic surgery_ = 0.99), but a higher death probability for horses with a history of recurrent colic (P_Recurrent colic > No colic_ = 0.87). Finally, horses that underwent laparotomy after NSS ablation had a greater death probability than those that did not require it (P_Surgery after NSS ablation > No Surgery after NSS ablation_ = 1) (Figure 4).

**Figure 5:**
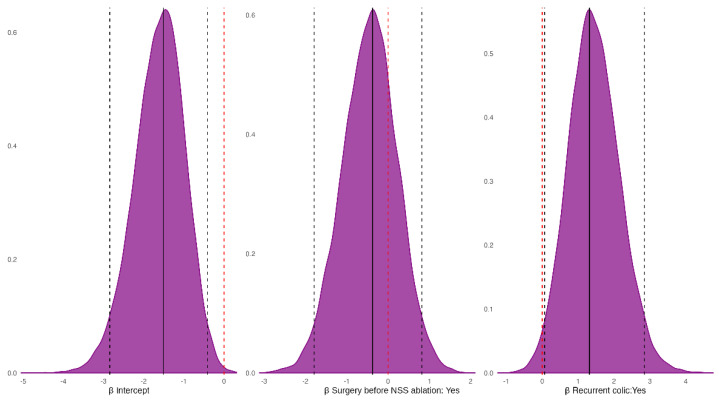
Posterior distributions of the fixed-effect coefficients from the Bayesian model for the probability of postoperative complications. Black solid vertical lines mark the half-sample mode (HSM); black dashed vertical lines mark the 95% highest-density intervals (HDI_95_); the red dashed vertical line marks zero.

The horses that underwent colic surgeries before NSS ablation had a negative effect on the onset of postoperative complications (P_Prior colic surgery < No colic surgery_ = 0.75), while a history of recurrent colic had a positive effect (P_Recurrent colic > No colic_ = 0.98) (Figure 5).

## 4. Discussion

Although laparoscopic NSS ablation has been described for over two decades, published data remain limited by small case numbers and heterogeneous follow-up. The present study adds contemporary outcome data from a cohort of 48 horses undergoing laparoscopic NSS ablation after LDDLC, with extended follow-up and the integration of time-to-event analyses.

Overall, short-term outcomes were favourable. Most horses were discharged without complications, supporting previous reports that standing laparoscopic NSS closure is a reliable procedure with low perioperative morbidity. In this cohort, NSS ablation was performed almost exclusively using self-retaining barbed sutures, reflecting the evolution from conventional intracorporeal knot tying and mesh-based techniques [2,24].

Long-term survival and return to athletic or reproductive activity were encouraging. However, recurrence of LDDLC was reported in 18.8% of horses, and other types of colic developed in 10.4%. While NSS ablation aims to prevent NSE, it does not exclude the occurrence of other forms of colic, which remain multifactorial [24,42]. Previous studies have likewise highlighted that colic episodes may still occur after closure, even when classical NSE or recurrent LDDLC is reduced [2,30]. From a clinical perspective, this supports the need for ongoing post-discharge monitoring, particularly in horses with a history of recurrent colic episodes.

Interpreting recurrence warrants caution. In our cohort, only a minority of suspected recurrences were surgically confirmed, and most were classified based on clinical evaluation supported by rectal palpation and/or ultrasonography. This limitation is relevant because rectal palpation, although often informative, may be non-specific or technically challenging in some individuals, and ultrasonography alone may be insufficient to definitively confirm LDDLC without surgical exploration [24,43,44]. Moreover, the inability to visualise the left kidney is not pathognomonic for LDDLC, as it may also occur with other large colon displacements or marked tympany [11,17]. Importantly, no follow-up (control) laparoscopy was performed, so the long-term completeness and durability of closure, and the possible contribution of suture failure, could not be assessed. This differs from experimental or small clinical reports in which second-look laparoscopy or post-mortem evaluation documented bridging fibrous tissue and, in some cases, complete closure [8,23,45]. This diagnostic and follow-up uncertainty may partially explain differences in recurrence estimates across published series, including those reporting no diagnosed LDDLC within their follow-up window.

The demographic distribution of the horses in this study, with a predominance of geldings and Warmblood breeds, aligns with previous reports [8,30]. While this pattern may reflect differences in management and referral populations, an anatomical contribution has also been proposed, as larger-framed horses may have conformational features that increase the likelihood of NSS involvement in large colon displacement [2,8,30]. The proportion of Arabian horses in this cohort is likely influenced by local breed distribution in the referral area. Consistently, a history of recurrent colic was common, supporting the concept that repeated episodes may identify horses predisposed to recurrence and therefore considered for preventive closure [30,46]. Retrospective evidence also suggests that NSS closure may reduce recurrent colic compared with non-operated horses, although diagnostic certainty and follow-up completeness vary across studies [30].

In the present cohort, most horses had not undergone colic surgery before referral for NSS ablation, which is compatible with the elective, preventive use of this procedure in horses with recurrent medically managed episodes and in selected cases following a surgically corrected episode [2,30]. Post-discharge colic was reported in 31.2% of cases. Recurrence of LDDLC was confirmed via laparotomy in three horses (6.3%) and suspected based on rectal palpation and/or ultrasonography in six horses (12.5%). In this context, the high proportion of suspected (rather than surgically confirmed) recurrences should be interpreted in light of the diagnostic and follow-up limitations outlined above, and it may partially explain why recurrence estimates differ across published series, including those reporting no diagnosed LDDLC after closure within their available follow-up window [30].

Time-to-event and Bayesian analyses provided clinically meaningful insights into prognosis. Horses requiring laparotomy during follow-up had markedly reduced survival, indicating that subsequent colic surgery identifies a high-risk subgroup, likely reflecting severe recurrent colic and/or major post-discharge complications. In this context, early recognition of deterioration and timely escalation of care may be particularly important. A history of recurrent colic was consistently associated with poorer outcomes and a higher probability of post-discharge complications, which is clinically plausible given that repeated episodes may reflect persistent predisposing factors and a more complex gastrointestinal history. By contrast, prior colic surgery showed a trend towards improved survival and fewer complications, but this apparently counterintuitive direction should be interpreted cautiously because it may reflect case selection and management factors rather than a true protective effect (for example, a healthy survivor effect, closer veterinary follow-up, or earlier escalation to definitive management).

Owner-reported satisfaction was high and broadly consistent with the high rate of functional return. This suggests that, for many owners, clinical improvement and functional recovery outweighed the occurrence of post-discharge events in determining overall perception of the procedure.

Several limitations should be considered. The retrospective design and the limited number of death events reduce statistical power and increase susceptibility to residual confounding. Follow-up duration was heterogeneous, and long-term information relied on telephone interviews, which may introduce recall bias and incomplete clinical characterisation of post-discharge colic episodes. Many suspected recurrences were not surgically confirmed, and the lack of follow-up laparoscopy prevented objective assessment of closure durability and the contribution of potential suture failure.

Despite these limitations, the present data support laparoscopic NSS ablation as a safe and clinically useful intervention in horses considered at risk of recurrent LDDLC, with favourable short-term outcomes and a high likelihood of functional return. Prospective studies with standardised diagnostic criteria, structured follow-up, and, where feasible, objective assessment of NSS closure would be valuable to better quantify true recurrence after NSS ablation and to distinguish LDDLC from other causes of post-discharge colic.

## 5. Conclusions

This retrospective cohort study supports laparoscopic NSS ablation as a safe and clinically useful intervention for horses considered at risk of recurrent left dorsal displacement of the large colon. Short-term outcomes were favourable, with high procedural success and a low frequency of in-hospital complications, and most horses were discharged in good clinical condition. Long-term follow-up indicated that most horses were alive at last contact and that functional recovery was common, as reflected by a high rate of return to athletic or reproductive activity and predominantly positive owner-reported satisfaction.

Nonetheless, post-discharge colic episodes were frequently reported, and recurrence of LDDLC was reported in nearly one-fifth of cases, although confirmation was limited and many suspected recurrences relied on clinical assessment supported by rectal palpation and/or ultrasonography. Time-to-event analyses indicated that laparotomy during follow-up was strongly associated with reduced survival, identifying a high-risk subgroup for which close monitoring and timely escalation of care are particularly important. A history of recurrent colic was consistently associated with poorer prognosis and a higher probability of postoperative complications, whereas prior colic surgery showed a non-significant trend towards improved survival and a negative association with postoperative complications that should be interpreted cautiously.

Prospective studies with standardised diagnostic criteria, structured follow-up, and objective assessment of the durability of NSS closure are warranted to better quantify true recurrence after NSS ablation and to distinguish LDDLC from other causes of post-discharge colic.

## Data Availability

The data presented in this study are available on request from the corresponding author.
